# Development of Thiophene Compounds as Potent Chemotherapies for the Treatment of Cutaneous Leishmaniasis Caused by *Leishmania major*

**DOI:** 10.3390/molecules23071626

**Published:** 2018-07-04

**Authors:** Felipe Rodriguez, Eva Iniguez, Guadalupe Pena Contreras, Haidar Ahmed, Thadeu E. M. M. Costa, Rachid Skouta, Rosa A. Maldonado

**Affiliations:** 1 Department of Biological Sciences, Border Biomedical Research Center, The University of Texas at El Paso, El Paso, TX 79968, USA; frodriguez16@miners.utep.edu (F.R.); eainiguez@miners.utep.edu (E.I.); 2 Department of Chemistry, Border Biomedical Research Center, The University of Texas at El Paso, El Paso, TX 79968, USA; gpenacontreras@miners.utep.edu (G.P.C.); hahmed2@miners.utep.edu (H.A.); 3 Centro de Desenvolvimento Tecnológico em Saúde, Fundação Oswaldo Cruz, Rio de Janeiro, RJ 21040-361, Brazil; temmcosta@gmail.com; 4 Instituto de Tecnologia em Fármacos-Farmanguinhos, Fundação Oswaldo Cruz, Rio de Janeiro, RJ 22775-903, Brazil; 5 Department of Biology, University of Massachusetts, Amherst, MA 01003-9297, USA

**Keywords:** thiophene compounds, *Leishmania major*, cutaneous leishmaniasis, drug screening, chemotherapy, structure–activity relationship (SAR), in silico docking, reactive oxygen species

## Abstract

*Leishmania major* (*L. major*) is a protozoan parasite that causes cutaneous leishmaniasis. About 12 million people are currently infected with an annual incidence of 1.3 million cases. The purpose of this study was to synthesize a small library of novel thiophene derivatives, and evaluate its parasitic activity, and potential mechanism of action (MOA). We developed a structure–activity relationship (SAR) study of the thiophene molecule **5A**. Overall, eight thiophene derivatives of **5A** were synthesized and purified by silica gel column chromatography. Of these eight analogs, the molecule **5D** showed the highest in vitro activity against *Leishmania major* promastigotes (EC_50_ 0.09 ± 0.02 µM), with an inhibition of the proliferation of intracellular amastigotes higher than 75% at only 0.63 µM and an excellent selective index. Moreover, the effect of **5D** on *L. major* promastigotes was associated with generation of reactive oxygen species (ROS), and in silico docking studies suggested that **5D** may play a role in inhibiting trypanothione reductase. In summary, the combined SAR study and the in vitro evaluation of **5A** derivatives allowed the identification of the novel molecule **5D**, which exhibited potent in vitro anti-leishmanial activity resulting in ROS production leading to cell death with no significant cytotoxicity towards mammalian cells.

## 1. Introduction

Leishmaniasis is a devastating neglected tropical disease (NTD) [1] caused by the protozoan parasite of the genus *Leishmania*. The parasite is transmitted from animals to humans through the bite of infected females *Lutzomyia* or *Phlebotomus* sand flies [2]. Over 20 species and subspecies of *Leishmania* infect humans, causing three major clinical forms of the disease: cutaneous (CL), visceral, and mucocutaneous leishmaniasis [3]. The prevalence of CL, the most common form of leishmaniasis, is estimated between 0.7 and 1.3 million new annually cases worldwide [4], and it is commonly caused by *Leishmania major* (*L. major*) or *L. mexicana.* CL presents as singular ulcerative or nodular lesions at the bite site that may resolve into scar tissue, often leading to scarring and social stigma [5]. The disease is present in both the Old World in regions of the Middle East, Africa, Central Western and Easter Europe; and the New World in regions of Central and South America, and more recently in North America [5].

Currently, there is no preventative or therapeutic human vaccine available against any clinical manifestation of the disease, and available treatments such as pentavalent antimonials (Glucantime and Pentostam), liposomal amphotericin B (AmBisome^®^), and miltefosine (IMPAVIDO^®^) present several disadvantages [6]. Pentavalent antimonial treatments are the first line of action, however, systemic therapy its required for more than 20 days, with toxic side effects including cardiotoxicity and hepatotoxicity [7,8]. Amphotericin B is highly active, but has extensive toxicity complications (nausea, vomiting, rigors, fever, hypertension or hypotension, and hypoxia) that usually lead to treatment interruption; besides, its administration requires hospitalization and its high cost limits its use in developed countries [5,9]. Miltefosine is the only oral agent against leishmaniasis, however, it presents several limitations such as embryo-fetal toxicity, fetal death, and its long half-life (150 h) may facilitate the emergence of drug resistance [10,11]. Thus, these facts clearly emphasize the urgent priority for the development of novel chemotherapies against leishmaniasis.

Considering the current interest in the search of antileishmanial agents, we previously reported for the first time that arylalkylamine type-compounds exhibit anti-*Leishmania* activity with no toxicity to mammalian cells [12,13]. Toward our medicinal chemistry effort in developing novel compounds with anti-parasitic activity and drug like properties (e.g., improved solubility, potency, stability and less or low toxicity), we assessed the antileishmanial activity of a series of novel compounds based on the thiophene scaffold with pharmaceutical properties: low toxicity, improved potency and solubility [13]. In this context, in the present study, we evaluated nine synthetic thiophene molecules derivatives against *L. major*.

We set a goal to design and synthesize a scaffold with antileishmanial activity in a one- or two-step synthesis using simple and efficient chemical transformations with high yield, high atom economy and inexpensive starting material. In this study, we focused on the creation of substituted thiophenes which are considered among the privileged structures in drug discovery [14]. Substituted thiophenes are known with their various biological activities such as anti-microbial, anti-cancer, and anti-inflammatory properties [14]. Therefore, the development of novel thiophene compounds with activity against *Leishmania* is crucial and urgent, as they may also complement current drugs and overcome drug resistance.

## 2. Results

### 2.1. Synthetic Chemistry

**Figure 1:**
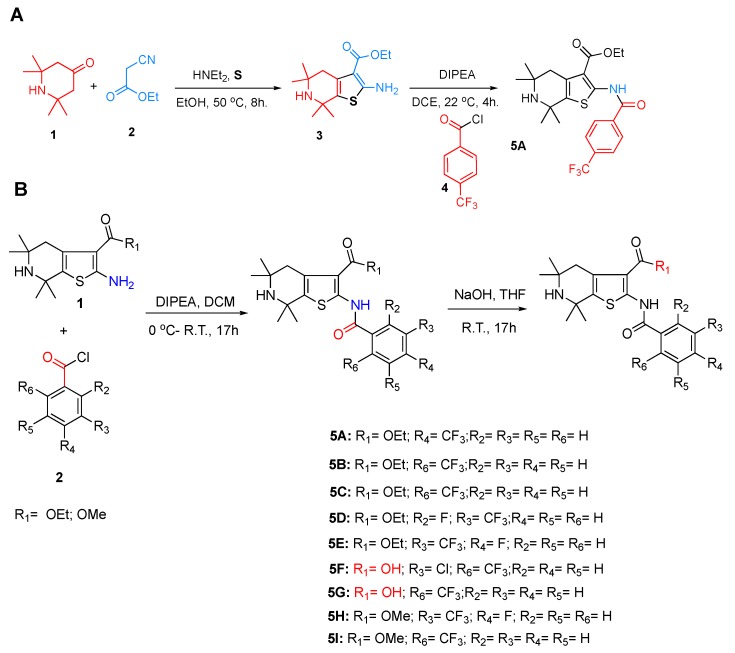
(**A**) Synthesis of thiophene **5A** (Ethyl 5,5,7,7-tetramethyl-2-(4-(trifluoromethyl)benzamido)-4,5,6,7-tetrahydrothieno [2,3-c]pyridine-3-carboxylate). (**B**) Synthetic route for the creation of thiophene compounds.

**Figure 2:**
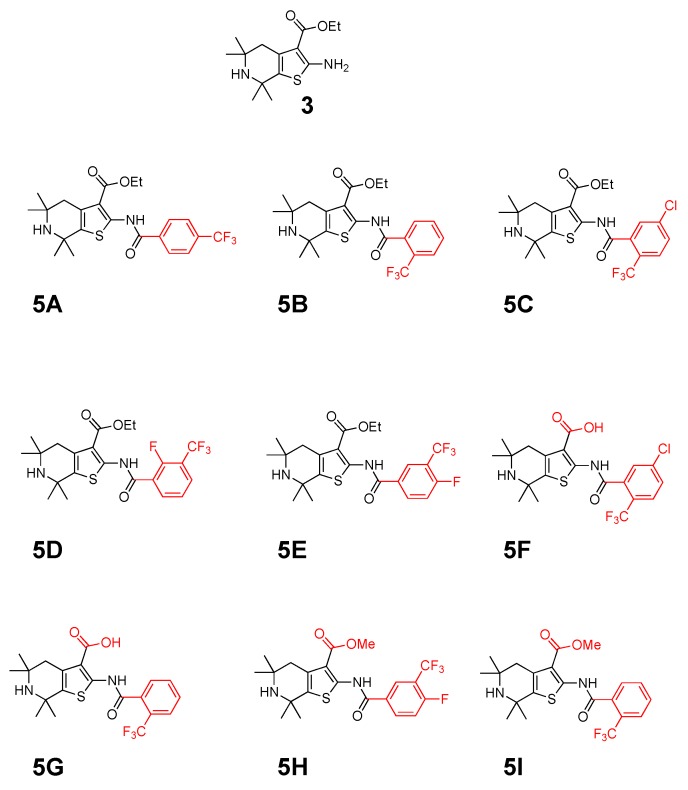
Chemical structures of the nine thiophene compounds.

Toward synthesizing the substituted thiophene **5A** efficiently, we used the well establish three components coupling reaction between a ketone, cyanoacetate and elemental sulfur [15] followed by a simple acylation reaction. The synthesis of **5A** is depicted in Figure 1A. After synthesizing **5A**, we developed the Structure–Activity Relationship (SAR) of **5A** and created eight analogs, as shown in Figure 1B. The purity of each analog was confirmed by ^1^H-NMR, ^13^C-NMR and MS, and then novel thiophene-like library compounds (Figure 2) were assessed for their potential in vitro antileishmanial activity.

### 2.2. Efficacy and Cytotoxicity of Parent Compound ***5A***

**Figure 3:**
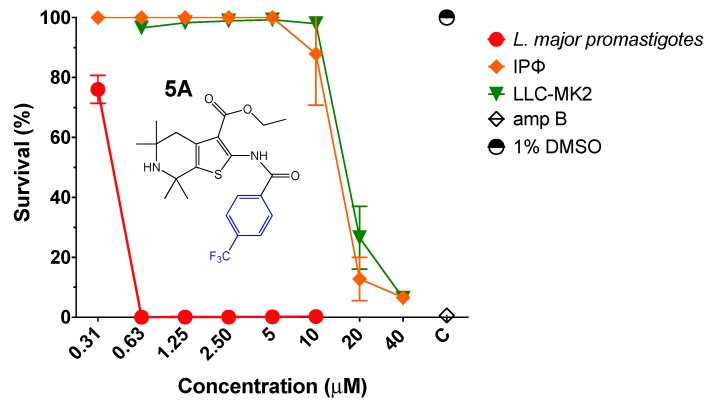
Antiparasitic effect of thiophene derivative **5A**. Viability of *L. major-luc* promastigotes incubated with **5A** at 0.31 to 10 µM for 72 h. Evaluation of intraperitoneal mouse macrophages (IPΦ) cytotoxicity for 48 h, or monkey kidney cells (LLC-MK2) treated with **5A** at concentrations of 0.31 to 40 µM for 72 h. Controls treated with 1% DMSO, or amphotericin B (amp B) at 5 µM.

First, the efficacy of thiophene derivative **5A** was evaluated against transgenic *L. major* promastigotes expressing firefly luciferase (*L. major-luc*) as previously described [16,17]. Compound **5A** at a range of 0.31–10 µM was incubated with 2 × 10^6^ *L. major-luc* promastigotes per mL for 72 h at 28 °C, and in vitro parasite viability (% survival) was measured by luciferase activity. Compound **5A** displayed an approximate 50% effective concentration (EC_50_) of 0.34 µM against *L. major* promastigotes (Figure 3 and Table 1). Furthermore, we investigated the potential cytotoxicity of **5A** by alamarBlue™ Cell Viability Assay (Thermo Fisher Scientific, Waltham, MA, USA) [18] and **5A** displayed a selective index (S.I.) of 30.58 against BALB/c intraperitoneal mouse macrophages (IPФ), and 51.91 in monkey kidney cells (LLC-MK2) (Table 1). 

### 2.3. In Vitro Anti-Leishmanial Activity of Thiophene Derivatives and Their Cytotoxicity

**Figure 4:**
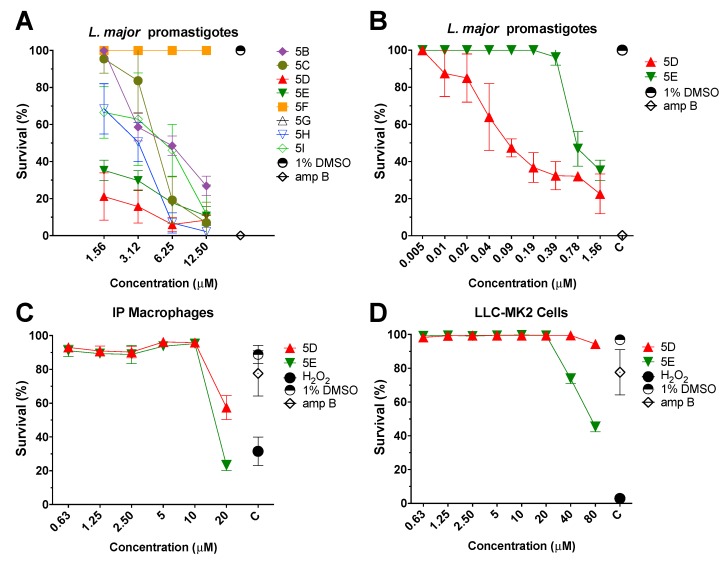
(**A**) Antiparasitic effect of the eight thiophene derivatives. Viability of *L. major-luc* promastigotes treated with the eight thiophene derivatives at a concentration of 1.56 to 12.5 µM for 72 h. (**B**) Evaluation of *L. major-luc* promastigotes treated with **5D** or **5E** thiophene derivatives at lower concentrations (0.005 to 1.56 µM) for 72 h. Controls treated with 1% DMSO, or amphotericin B (amp B) at 5 µM. (**C**) Cytotoxicity evaluation of intraperitoneal mouse macrophages (IPΦ) treated with **5D** or **5E** thiophene derivatives at concentrations from 0.63 to 20 µM for 48 h. (**D**) Cytotoxicity evaluation of monkey kidney cells (LLC-MK2) treated with **5D** or **5E** thiophene derivatives at a concentrations from 0.63 to 80 µM for 72 h. Controls treated with 1% DMSO, amp B at 5 µM, or 5% of hydrogen peroxide (H_2_O_2_). Cells were stained with Hoechst 33342 (healthy cell) and Propidium Iodide (compromised cell)*.*

Consequently, to lower the toxicity and increase the parasitic activity, eight new thiophene molecules (Figure 2) were evaluated. First, the thiophene compounds were tested in the presence of increasing drug concentrations (1.56–12.5 µM), followed by incubation with *L. major-luc* promastigotes (2 × 10^6^/mL) for 72 h at 28 °C. The experiment was performed using the same conditions as described for the parent drug **5A**. As summarized in Table 1, all eight thiophene molecules showed promising antileishmanial activity against *L. major* promastigotes with an EC_50_ ranging from 0.09 to 6.25 µM (Figure 4A). However, the best thiophene compounds were **5D** (EC_50_ 0.09 ± 0.02 µM) and **5E** (EC_50_ 0.78 ± 0.11 µM) (Figure 4B and Table 1). 

Consequently, cytotoxicity assays were performed by incubating LLC-MK2 or IPФ with compounds. First, **5D** or **5E** were incubated with 1 × 10^5^ LLC-MK2/mL or 1 × 10^5^ IPФ/mL for 72 and 48 h, respectively. Interestingly, compound **5D** did not display perceptible toxicity against LLC-MK2 at concentrations up to 80 µM (Figure 4C,D). In the case of IPФ, **5D** exhibited a CC_50_ value of 27.89 ± 3.19 µM and an excellent S.I. of 310. As **5E** compound CC_50_ values of 80 ± 4.45 µM in LLC-MK2 cells and 16.59 ± 1.52 µM in IPФ, with S.I. values of 102.56 and 21.27, respectively. More importantly, we determined that both **5D** and **5E** displayed lower cytotoxicity to mammalian cells and higher parasitic activity than parent compound **5A** (Table 1). 

### 2.4. In Vitro Efficacy of Thiophene ***5D*** Against Intracellular Amastigotes

**Figure 5:**
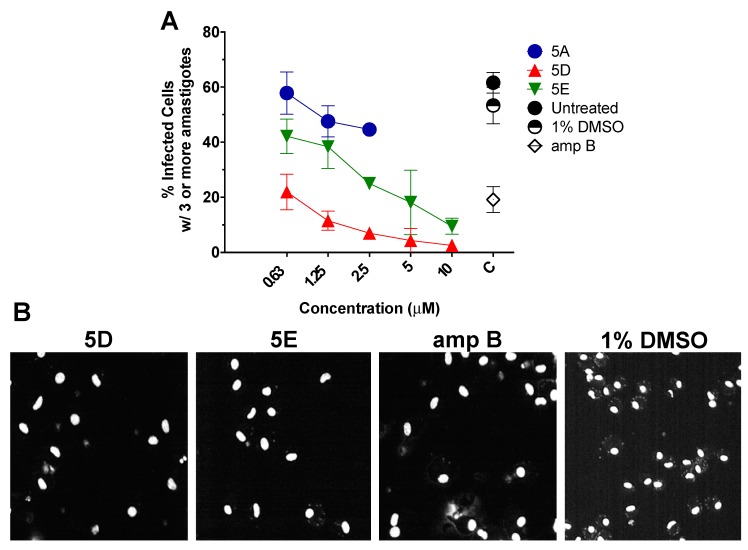
(**A**) High content imaging assay (HCIA) analysis of intraperitoneal mouse macrophages (IPΦ) infected with *L. major-luc* amastigotes, followed by treatment with **5D** or **5E** from 0.63 to 10 µM for 48 h. Controls included untreated, 1% DMSO, amphotericin B (amp B) at 5 µM, or parent drug **5A**. Data are represented as the percentage (%) of infected IPΦ with three or more amastigotes per cell. Note: Data for **5A** at concentration 5 and 10 µM were not generated because **5A** was cytotoxic for IPΦ at such concentration. (**B**) Representative monochromatic images of infected IPΦ with *L. major* after 48 h treatment with **5D** or **5E** at 2.5 µM, amp B (5 µM), or 1% DMSO.

Additionally, efficacy of thiophene compounds **5D** and **5E** was tested against the infectious intracellular amastigote form of *L. major*, by High-Content Imaging Assay (HCIA) on infected intraperitoneal mouse macrophages. As observed in Figure 5A, in comparison with untreated control and 1% DMSO, **5D** and **5E** inhibited the proliferation of the intracellular amastigotes by more than 75% and 50%, respectively, at a 0.625 µM concentration. Furthermore, as observed in Figure 5B, a reduced number of infected cells were observed after **5D** or **5E** treatment (2.5 µM) when compared to control treated with 1% DMSO.

### 2.5. Molecule ***5D*** Induces ROS in L. major

**Figure 6:**
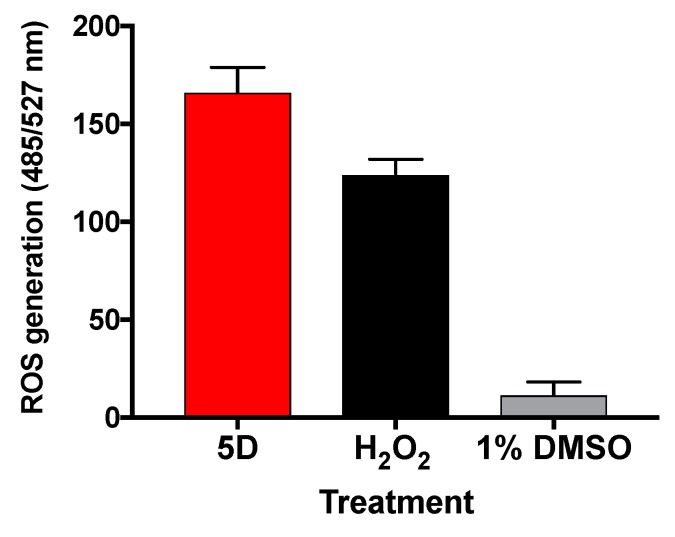
Generation of reactive oxygen species in *L. major* treated for 31 h with **5D** at 0.90 µM (EC_50_). Values shown are the mean and standard error of five different replicates minus basal fluorescence. Control treated with 1% DMSO or hydrogen peroxide (H_2_O_2_) at 100 µM.

Based on our previous study [12], it was hypothesized that **5D** may induce parasite death through the production of ROS. Thus, 2 × 10^6^ *L. major* promastigotes per mL were incubated with **5D** (EC_50_ 0.09 ± 0.02 µM). After 24 h, ROS levels were measured by the addition of 10 µM of the cell-permeable dye H_2_DCFDA (Thermo Fisher Scientific, Waltham, MA, USA), and fluorescence was monitor for an additional 7 h using a fluorometer. As expected, ROS levels in **5D** treated parasites were 14.5-fold higher compared to vehicle control 1% DMSO (Figure 6).

### 2.6. Docking of ***5D*** on TryR from Leishmania

Next, to determine the possible molecular mechanism responsible for the antileishmanial activity of **5D**, docking studies on TryR from *L. infantum* (PDB id: 2JK6) were performed. Using Glide Standard Precision [19] and Extra Precision (XP), we performed Rigid Receptor Docking analysis of control (Quinacrine Mustard) and **5D**. The 3D ligand structures were docked against the best potential binding site of 2JK6. Glide SP and XP only accounts for the ligand being dynamic however the protein remains rigid. Docking box coordinates and dimensions remained all at default (20 × 20 × 20 Å). Glide XP gives an output of a docking score, which was analyzed by the lowest number, or whichever is more negative to be the highest scoring ligand. The docking results, summarized in Table 2, showed the control (Quinacrine Mustard) with higher binding affinity than **5D** in both SP and XP. However, the XP docking score did not differ by much, indicating more rigorous docking analysis is needed. Thus, both ligands were taken to Schrodinger’s Flexible receptor docking.

Schrodinger IFD protocol for all IFD jobs was used [20]. The IFD program makes use of both Glide (for docking) and Prime (for protein structure modeling). The combination of the two software packages allows a more accurate ligand binding calculation. We performed the re-docking with Glide XP for the refined docking results [21]. The IFD data presented in Table 2 show that our lead molecule **5D** had a better binding affinity. Docking scores from Rigid Receptor Docking and Flexible Receptor

**Figure 7:**
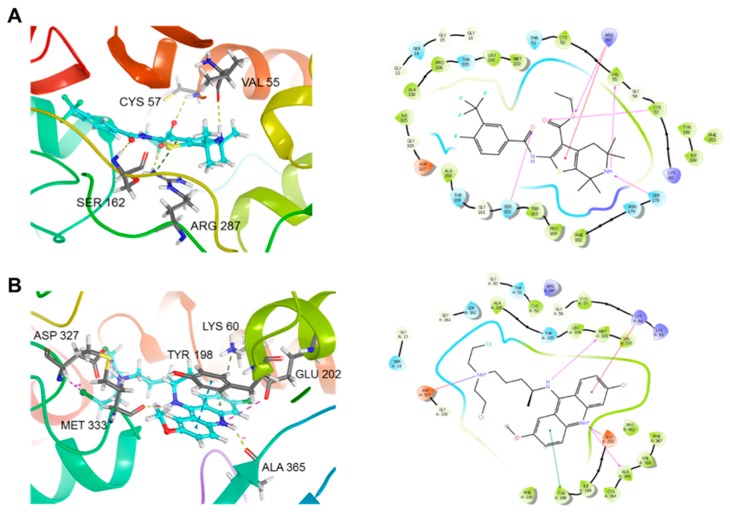
Trypanothione Reductase from *L. infantum* (PDB: 2JK6) Protein–ligand complex of Induced Fit Docking (IFD): (**A**) ligand interaction diagram of **5D** in complex with 2JK6 and 2D ligand interaction and; (**B**) ligand interaction diagram of Quinacrine Mustard in complex with 2JK6 and 2D ligand interaction.

Docking differed significantly. This is accounted for the protein dynamic movement during drug binding in IFD. Furthermore, Figure 7A,B presents the IFD binding pocket of the protein–ligand complex. Figure 7A shows our lead molecule **5D** which displays hydrogen bond interactions with SER 1632, ARG 287, VAL 55, and also with CYS 57. Compound **5D** also exhibits π-cation interaction with residue ARG 287. Quinacrine Mustard interacted with a new set of residues and showed only two hydrogen bonds between MET 333 and ALA 365 (Figure 7B). The control also formed salt bridges with ASP 327 as well as GLU 202. π–π and π–cation interaction was also shown between TYR 198 and LYS 60, respectively. These results provided evidence that the possible MOA of **5D** may be through the inhibition of TryR, an essential enzyme to the thiol metabolism of the parasite [22,23], and promising chemotherapeutic target against leishmaniasis [24].

## 3. Discussion

There is an urgent need for new therapeutics that are more effective and less toxic than conventional treatments used to treat infectious diseases, including leishmaniasis [25]. Thiophenes derivatives are known for their therapeutic applications and have shown promising results to treat different types of cancer, degenerative diseases, HIV, and malaria [26,27,28,29,30,31,32,33]. Thus, we evaluated the anti-leishmania activity and selectivity of nine thiophene derivatives against *L. major*, and potential MOA was elucidated for our best candidate, **5D**.

Thiophene derivatives **5A**, **5D** and **5E** exhibited potent parasitic activity against *L. major* promastigotes (Table 1). Experimental models involving macrophages are ideal to study leishmaniasis since they are the major host cell for *Leishmania* spp. [34]. Thus, our three best candidates were further evaluated against the most important form of the parasite, intracellular amastigotes, in an in vitro infection model of murine macrophages. In this case, **5D** presented the best anti-leishmanial activity by decreasing the proliferation of the parasite by 80%.

The in vitro toxicity of **5D** and **5E** was evaluated towards IPФ and LCC-MK2 cells. Our best two compounds were safer for the two cytotoxic models than the reference drug, amphotericin B, which is already known for its cytotoxic effects [35]. Even though amphotericin B presented similar activity as **5D** against promastigotes and amastigotes, this result further supports the application of **5D** and **5E** as anti-leishmanial agents. Furthermore, the selectivity presented by **5D** was remarkably higher than the parent compound **5A** (10-fold higher), demonstrating the success to increase the anti-leishmanial activity and reduced cytotoxicity effects when compared to our previously reported arylalkylamine type-compound [12].

Next, we studied the potential MOA of derivative **5D**. ROS can be generated in response to some drugs, resulting in destruction of cellular macromolecular components inducing cell death by affecting parasite mitochondrial function [36,37]. Here, we observed that **5D** induced ROS production in *L. major* promastigotes after 31 h of treatment. The redox homeostasis in *Leishmania* is achieved through the activity of several superoxide dismutases, heme peroxidases, as well as of a series of thiol-containing proteins that directly or indirectly depend on trypanothione reductase [23,38]. In this regard, the trypanothione metabolism is unique to trypanosomatids and its main detoxification pathway [39]. This pathway protects parasites from oxidative stress and participates in several cellular processes that are carried out by glutathione in other organisms. Moreover, there are several trypanothione-dependent pathways that include enzymes such as tryparedoxin peroxidase (detoxication of hydroperoxide), ascorbate peroxidase (homeostasis of ascorbate), ribonucleotide reductase (synthesis of DNA precursors), and others [40,41]. With this idea on mind, we decided to explore in silico docking analysis to assess the possibility of *L. major* TryR as the target of **5D**. Moreover, our results suggested that TryR interacts with **5D**, however we do not exclude the possibility that other redox metabolism enzymes could be also targeted by compound.

In conclusion, to discover new chemotherapy agents against leishmaniasis, we efficiently synthetized nine thiophene type-compounds including **5A**, following a two-step synthesis from low-priced commercially available starting materials. We then showed that our novel thiophene type-compounds possess high in vitro antileishmanial activity. Based on our SAR study, **5D** analog was selected as the most promising lead compound among this library with excellent antiparasitic and S.I. Furthermore, **5D** may act against trypanothione metabolism, followed by the production of ROS in the parasite; nevertheless, biological studies with recombinant TryR enzyme needs to be performed to further support this assumption. Overall, **5D** represents a potential chemotherapeutic agent for the treatment of leishmaniasis, and further evaluation in a pre-clinical mouse model of cutaneous leishmaniasis is currently in progress in our laboratory.

## 4. Materials and Methods 

### 4.1. General

Unless otherwise noted, all commercial reagents were used as purchased from Aldrich, St. Louis, MO, USA. All the reactions were monitored by thin-layer chromatography (TLC) that was performed on silica gel plates GF254. Compounds were visualized under a UV lamp. Flash chromatography was performed using silica gel (200–300 mesh) with various ratios of Dichloromethane: Methanol solvents as indicated in the text. Spectroscopy: ^1^H and ^13^C-NMR spectra were obtained on a Bruker DPX 400 MHz. Chemical shifts (δ) are quoted in parts per million (ppm), to the nearest 0.01 ppm and internally referenced relative to the solvent nuclei. ^1^H-NMR spectral data are reported with their chemical shift in parts per million (ppm). The multiplicity in ^1^H-NMR is abbreviated as follows: brs: broad; s: singlet; d: doublet; t: triplet; q: quartet; quint: quintet; sext: sextet; *m:* multiplet; or as a combination (e.g., dd, dt, etc.). The coupling constant (*J*) in hertz, integration and proton count were determined.

Spectrometer: Liquid chromatography/mass spectra (LC-MS) [+ESI] were taken on double focusing sector type mass spectrometer HX-110A. Maker JEOL Ltd. Tokyo, Japan (resolution of 10,000 and 10 KV accel. Volt. Ionization method; FAB (Fast Atom Bombardment) used Xe 3 KV energy. Used Matrix, NBA (m-Nitro benzyl alcohol)). Melting points were measured using a Mel-Temp melting point apparatus.

### 4.2. Chemical Synthesis

#### 4.2.1. General Synthetic Procedure 1

##### Synthesis of the Ethyl 5,5,7,7-tetramethyl-2-(4-(trifluoromethyl) benzamido)-4,5,6,7-tetrahydrothieno[2,3-c]pyridine-3-carboxylate (***5A***)

Briefly, the 2,2,6,6-tetramethylpiperidine ketone (**1**, 1 equivalent) was mixed with 2-cyanoacetate esters (**2**, 1 equivalent) and elemental sulfur (1 equivalent) in the presence of diethylamine (3 equivalents) in ethanol (ETOH) at 60 °C for 17 h. The crude was precipitated by adding water and filtered to provide the desired known 2-aminothiophene intermediate (**3**) [12]. The latter was reacted with 4-trifluomethy benzoyl chloride in the presence of diisopropylethylamine in dry dichloromethane at 0 °C for 1 h and then at room temperature for 4 h. The solvent was evaporated then the crude mixture was purified by flash-column chromatography on silica gel, using a mixture of solvent of dichloromethane: methanol (DCM: MeOH) at ratios from 100:1 to 50:1, to provide the desired ethyl 5,5,7,7-tetramethyl-2-(4-(trifluoromethyl)benzamido)-4,5,6,7-tetrahydrothieno [2,3-c]pyridine-3-carboxylate (**5A**) as a light brown solid. The purity of **5A** was confirmed by ^1^H-NMR, ^13^C-NMR, LC/MS and melting point.

#### 4.2.2. General Synthetic Procedure 2

##### Synthesis of 5,5,7,7-tetramethyl-2-(2-(trifluoromethyl)benzamido)-4,5,6,7-tetrahydrothieno [2,3-c]pyridine-3-carboxylic acid (***5G***)

To ethyl 5,5,7,7-tetramethyl-2-(2-(trifluoromethyl)benzamido)-4,5,6,7-tetrahydrothieno [2,3-c]pyridine-3-carboxylate compound (**5B**; 1 equiv.) in 1 mL of tetrahydrofuran (THF), was added 5 equiv. of sodium hydroxide (NaOH) in water (1 mL). The mixture was stirred at room temperature during 17 h. The THF solvent was evaporated then mixture was acidified with HCl 1M to pH = 4. The protonated acid compound was extracted with ethylacetate (EtOAc) (3×; 50 mL) then dried under magnesium sulfate (MgSO_4_), and the solvent was evaporated to provide the desired 5,5,7,7-tetramethyl-2-(2-(trifluoromethyl)benzamido)-4,5,6,7-tetrahydrothieno [2,3-c]pyridine-3-carboxylic acid (**5G**), (Figure 2). The purity of **5G** analog was confirmed by ^1^H-NMR, ^13^C-NMR, and LC/MS.

Following the general synthetic procedure 1, and in the presence of various substituted benzoyl chlorides, we created the analogs **5B**–**5E**, and **5H**–**5I**.

##### Ethyl-5,5,7,7-tetramethyl-2-(2-(trifluoromethyl)benzamido)-4,5,6,7-tetrahydrothieno[2,3-c]pyridine-3-carboxylate (***5B***)

Brown solid, yield 75%, m.p. 181.5–182.5 °C. ^1^H-NMR (400 MHz, CDCl_3_) δ 11.68 (s, 1H, NHCO), 7.78–7.65 (m, 4H, ArH), 4.32 (q, *J* = 7.0 Hz, 3H, CH_2_), 2.70 (s, 2H, CH_2_), 1.92 (m, 1H, NH), 1.39–1.16 (m, 15H, 5 × CH_3_). ^13^C-NMR (101 MHz, CDCl_3_) δ 166.18, 163.70, 147.33, 135.09, 133.90, 132.18, 130.71, 128.50, 128.32, 126.68, 112.30, 60.61, 51.89, 49.84, 39.53, 34.13, 29.99, 14.00. LC-MS (ESI) for C_22_H_25_F_3_N_2_O_3_S, M + 1 = 455.22.

##### Ethyl-5,5,7,7-tetramethyl-2-(2-(trifluoromethyl)benzamido)-4,5,6,7-tetrahydrothieno[2,3-c]pyridine-3-carboxylate (***5C***)

Brown solid, yield 70%, m.p. 132.6–133.6 °C. ^1^H-NMR (400 MHz, CDCl_3_) δ 11.56 (s, 1H, NHCO), 7.52–7.40 (m, 3H, ArH), 4.10 (q, *J* = 7.1 Hz, 3H, CH_2_), 2.53 (s, 2H, CH_2_), 1.32–1.03 (s, 15H, 5 × CH_3_). ^13^C-NMR (101 MHz, CDCl_3_) δ 166.05, 162.14, 146.93, 138.40, 135.50, 135.04, 130.73, 128.58, 128.43, 128.25, 126.30, 112.53, 60.69, 52.17, 50.17, 39.27, 33.88, 29.71, 13.96. (ESI+, M + 1 = 489.02. LC-MS (ESI) for C_22_H_24_ClF_3_N_2_O_3_S, M + 1 = 489.02.

##### Ethyl-2-(2-fluoro-3-(trifluoromethyl)benzamido)-5,5,7,7-tetramethyl-4,5,6,7-tetrahydrothieno[2,3-c]-pyridine-3-carboxylate (***5D***)

Light brown solid, yield 76%, m.p. 145.4–146.4 °C. ^1^H-NMR (400 MHz, CDCl_3_) δ 12.40 (d, *J* = 10.3 Hz, 1H, NHCO), 8.49–8.30 (m, 1H, ArH), 7.83 (dd, *J* = 10.5, 3.9 Hz, 1H, ArH), 7.45 (t, *J* = 7.8 Hz, 1H, ArH), 4.42 (q, *J* = 7.1 Hz, 2H, CH_2_), 2.74 (s, 2H, CH_2_), 1.54 (s, 6H, 2 × CH_3_), 1.42 (t, *J* = 7.1 Hz, 3H, CH_3_), 1.23 (t, *J* = 7.1 Hz, 6H, 2 × CH_3_). ^13^C-NMR (101 MHz, CDCl_3_) δ 165.70, 158.50, 146.67, 136.26, 135.74, 131.23, 129.15, 124.87, 123.48, 121.53, 119.30, 113.11, 60.86, 52.03, 50.01, 39.71, 34.34, 30.23, 14.29. LC-MS (ESI) for C_22_H_24_F_4_N_2_O_3_S, M + 1 = 473.05.

##### Ethyl-2-(4-fluoro-3-(trifluoromethyl)benzamido)-5,5,7,7-tetramethyl-4,5,6,7-tetrahydrothieno[2,3-c]-pyridine-3-carboxylate (***5E***)

Light brown solid, yield 66%, m.p. 116.0–117.0 °C. ^1^H-NMR (400 MHz, CDCl_3_) δ 12.40 (s, 1H, NHCO), 8.25 (dd, *J* = 6.4, 1.4 Hz, 1H, ArH), 8.10 (ddd, *J* = 7.9, 4.1, 2.2 Hz, 1H, ArH), 7.29 (t, *J* = 9.1 Hz, 1H, ArH), 4.34 (q, *J* = 7.1 Hz, 2H, CH_2_), 2.65 (s, 2H, CH_2_), 1.46 (s, 6H, 2 × CH_3_), 1.36 (t, *J* = 7.1 Hz, 3H, CH_3_), 1.16 (d, *J* = 11.3 Hz, 6H, 2 × CH_3_). ^13^C-NMR (101 MHz, CDCl_3_) δ 166.89, 160.81, 148.04, 135.17, 132.73, 128.95, 128.58, 127.43, 119.35, 117.73, 117.51, 112.28, 60.92, 53.37, 52.12, 50.08, 39.57, 34.13, 30.03, 14.18. LC-MS (ESI) for C_22_H_24_F_4_N_2_O_3_S, M + 1 = 473.05.

##### Methyl-2-(4-fluoro-3-(trifluoromethyl)benzamido)-5,5,7,7-tetramethyl-4,5,6,7-tetrahydrothieno[2,3-c]-pyridine3-carboxylate (***5H***)

Light brown solid, yield 72%, m.p. 122.0–123.0 °C. ^1^H-NMR (400 MHz, CDCl_3_) δ 12.45 (s, 1H, NHCO), 8.32 (s, 1H, ArH), 8.18 (s, 1H, ArH), 7.37 (t, *J* = 8.0 Hz, 1H, ArH), 3.93 (s, 3H, CH_3_), 2.71 (s, 2H, CH_2_), 1.53 (s, 6H, 2 × CH_3_), 1.27–1.16 (m, 9H, 3 × CH_3_).^13^C-NMR (101 MHz, CDCl_3_) δ 167.47, 161.06, 160.86, 148.44, 132.84, 132.74, 129.03, 128.46, 127.53, 117.86, 117.64, 112.17, 52.45, 51.80, 50.45, 39.49, 34.13, 29.98 LC-MS (ESI) for C_21_H_22_F_4_N_2_O_3_S, M + 1 = 459.07.

##### Methyl-5,5,7,7-tetramethyl-2-(2-(trifluoromethyl)benzamido)-4,5,6,7-tetrahydrothieno[2,3-c]pyridine-3-carboxylate (***5I***)

Light brown solid, yield 60%, m.p. 184.2–185.2 °C. ^1^H-NMR (400 MHz, CDCl_3_) δ^1^H-NMR (400 MHz, CDCl_3_) δ 11.67 (s, 1H, NHCO), 7.78 (d, *J* = 7.4 Hz, 1H, ArH), 7.66 (dd, *J* = 10.9, 4.5 Hz, 3H, ArH), 3.85 (s, 3H, CH_3_), 2.68 (s, 2H, CH_2_), 1.52 (s, 6H, 2 × CH_3_), 1.23 (s, 6H, 2 × CH_3_). ^13^C-NMR (101 MHz, CDCl_3_) δ 166.90, 164.03, 147.80, 135.24, 134.04, 132.27, 130.83, 128.57, 127.92, 126.90, 126.85, 112.18, 52.18, 51.64, 50.17, 39.59, 34.26, 30.12 LC-MS (ESI) for C21H23F3N2O3S, M + 1 = 441.07.

Following the general synthetic procedure 2, we created the analogs **5F** and **5G**.

##### 2-(5-chloro-2-(trifluoromethyl)benzamido)-5,5,7,7-tetramethyl-4,5,6,7-tetrahydrothieno[2,3-c]pyridine-3-carboxylic acid (***5F***)

Brown oilesh, yield 51% ^1^H-NMR (400 MHz, CDCl_3_) δ 11.69 (s, 1H, NHCO), 7.57–7.44 (m, 3H, ArH), 2.53 (s, 2H, CH_2_), 1.32–1.03 (s, 12H, 4 × CH_3_). ^13^C-NMR (101 MHz, CDCl_3_) δ 167.09, 164.14, 161.83, 139.04, 135.50, 135.04, 130.73, 128.58, 128.43, 128.25, 126.30, 112.53, 52.17, 50.17, 39.27, 33.88, 29.71. LC-MS (ESI) for C_22_H_24_ClF_3_N_2_O_3_S, M + 1 = 461.03.

##### 5,5,7,7-tetramethyl-2-(2-(trifluoromethyl)benzamido)-4,5,6,7-tetrahydrothieno[2,3-c]pyridine-3-carboxylic acid (***5G***)

Brown oilesh, yield 55%. ^1^H-NMR (400 MHz, CDCl_3_) δ 11.70 (s, 1H, NHCO), 7.92–7.62 (m, 4H, ArH), 2.70 (s, 2H, CH_2_), 1.39–1.16 (m, 12H, 4 × CH_3_). ^13^C-NMR (101 MHz, CDCl_3_) δ 167.08, 163.70, 162.03, 136.09, 134.01, 132.18, 130.71, 128.50, 128.32, 126.68, 112.30, 51.89, 49.84, 39.53, 34.13, 29.99. LC-MS (ESI) for C_22_H_25_F_3_N_2_O_3_S, M + 1 = 427.02.

### 4.3. Cell Maintenance

Transgenic *L. major* promastigotes expressing firefly luciferase (*L. major-luc*) (Lmj-FV1-LUC-TK [*L. major* strain Friedlin {MHOM/JL/80/Friedlin}] were maintain in M199 medium supplemented with hemin, 10% inactivated fetal bovine serum (iFBS) (Gibco, Thermo Fisher Scientific, Waltham, MA, USA), and 1% of 10,000 units/mL penicillin and 10 mg/mL streptomycin (Gibco, Thermo Fisher Scientific, Waltham, MA, USA) as described [42]. Intraperitoneal murine macrophages (IPФ) were obtained from BALB/c mice [43]. Monkey kidney epithelial cells (LLC-MK2) (ATCC # CCL-7) (American Type Culture Collection, Manassas, VA, USA), and IPФ were cultured in Dulbecco’s Modified Eagle’s Medium (DMEM), supplemented with 10% iFBS, along with 1% of 10,000 units/mL penicillin and 10 mg/mL streptomycin. The procedures were performed minimizing the distress and pain for animals following the NIH guidance and animal protocol (A-201107-1) approved by UTEP’s Institutional Animal Care and Use Committee (IACUC).

### 4.4. Luciferase Assay—Viability of Leishmania Major promastigotes

The antiparasitic activity of the 9 thiophene compounds was determined by adding the analogs together with 2 × 10^6^ *L. major-luc* promastigotes per mL in 96-well NUNC white microplates (Thermo Fisher Scientific, Waltham, MA, USA) followed by incubation for 72 h at 28 °C. Then, parasite survival was measured by luciferase activity with the addition of the substrate 5′-fluoroluciferin (ONE-Glo luciferase assay system; Promega, Madison, WI, USA), using a luminometer (Luminoskan; Thermo Fisher Scientific, Waltham, MA, USA). The luminescence intensity was a direct measure of the parasite survival, and 50% effective concentration (EC_50_) was determined for each drug and summarized in Table 1.

### 4.5. Assessment of Thiophene Compound Mammalian Cell Cytotoxicity 

The potential cytotoxicity of **5A** was tested by alamarBlue^TM^ Cell Viability Assay (Thermo Fisher Scientific, Waltham, MA, USA) as previously described [18]. Briefly, 1 × 10^6^/mL rhesus monkey kidney epithelial cells (LLC-MK2), and 1 × 10^6^/mL BALB/c IPФ were seeded in a 96-well clear bottom black microplate (BD Biosciences, Franklin Lakes, NJ, USA). Cells were incubated in the presence of increasing drug concentrations for 72 h (LLC-MK2) or 48 h (IPФ) at 37 °C, 5% CO_2_, followed by addition of alamarBlue^TM^. Fluorescence was measured using a fluorometer (Fluoroskan; Thermo Fisher Scientific, Waltham, MA, USA). Compound **5D** or **5E** were incubated with 1 × 10^5^ cells/mL (LLC-MK and IPФ) for 72 or 48 h, respectively, at 37 °C, 5% CO_2_. After the incubation period, a dilution of 20:1000 in PBS from a stock at 1 mg/mL of Propidium Iodide (PI) and Hoechst 33342 (Thermo Scientific, Waltham, MA, USA) were added for survival discrimination as previously described [44]. Analysis was performed by High-Content Imaging Assay (HCIA) using an IN Cell 2000 Analyzer Bioimaging System (GE Healthcare, Chicago, IL, USA) for LLC-MK2 cells, and BD Pathway 855 High-resolution Bioimager System (BD Biosciences, Franklin Lakes, NJ, USA) for IPФ. The 50% cytotoxic concentration (CC_50_) and selective index (S.I.) was determined and summarized in Table 1.

### 4.6. High-Content Imaging Assay—Proliferation Experiments

BALB/c IPФ were acquired and seeded at a density of 1 × 10^5^ cells/mL for 2 h at 37 °C, 5% CO_2_. After adherence, IPФ were infected with 1 × 10^6^/mL metacyclic promastigotes of *L. major-luc*, at a ratio of 10:1 parasites per macrophage. Subsequently, infected IPФ were incubated with derivatives **5A**, **5D** and **5E** at increasing concentrations (0.625 to 10 µM) for 48 h treatment. Afterwards, cells were fixed with 4% paraformaldehyde, and stained with (1.25:100) Alexa Fluor™ 488 Phalloidin (Thermo Fisher Scientific, Waltham, MA, USA) and (1:1000) DAPI (Sigma Aldrich, St. Louis, MO, USA). Then, the numbers of infected cells and amastigotes were determined by HCIA using an IN Cell 2000 Analyzer Bioimaging System (GE Healthcare, Chicago, IL, USA). Parameters were set for the excitation and emission spectra of Alexa Fluor™ 488 Phalloidin and DAPI, and a constraint of 3 or more parasites per macrophage was set as previously reported [44,45].

### 4.7. Measurement of Reactive Oxygen Species Levels

*L. major* promastigotes (2 × 10^6^ cells/mL) were incubated for 24 h with **5D** (EC_50_ 0.90 µM) in a 96-well clear bottom black microplate (BD Biosciences, Franklin Lakes, NJ, USA). Controls treated with 1% DMSO, 100 µM of hydrogen peroxide (H_2_O_2_) as positive control, or M199 medium. After incubation period, 10 µM of H_2_DCFDA (Thermo Fischer Scientific, Waltham, MA, USA) (H_2_DCFDA/DMSO, 1 mg/mL) was added per well followed by 20 min of incubation at 37 °C. Fluorescence was measured for an additional 7 h using a fluorometer (Fluoroskan; Thermo Fisher Scientific, Waltham, MA, USA) at 527 nm using an excitation wavelength of 485. For all measurements, basal fluorescence was subtracted. 

All graphs, EC_50_ and CC_50_ values were produced using Graph Pad Prism 7 Software (GraphPad Software, Inc., La Jolla, CA, USA). 

### 4.8. Docking Studies—Pre-Docking Preparation

The structure of trypanothione reductase bound to Flavin adenine dinucleotide (PDB ID: 2JK6) [46] were obtained from the Protein Data Bank. The Protein Preparation Wizard in Maestro was used to minimize the protein structure, add hydrogens and charges, and find any missing residues. The two-dimensional structures of **5D** and Quinacrine Mustard, a recently experimentally approved drug as a control, were drawn using the molecular structure editor ChemDraw Software (PerkinElmer, Waltham, MA, USA) and processed by LigPrep Schrödinger (Schrödinger, LLC., New York, NY, USA) to generate the 3D structures. 

### 4.9. Binding Site Analysis

Maestro’s SiteMap tool (Schrödinger, LLC., New York, NY, USA) was used to predict the likely binding sites of trypanothione reductase. The SiteMap tool uses a series of algorithm that generates a map of hydrophobic and hydrophilic surfaces on the protein surface [47]. Hydrophilic surface maps are divided into donor, acceptor, and metal-binding regions. Five potential binding sites were identified with at least 15 site points. However, the top SiteMap was chosen to be the receptor grid.

## Figures and Tables

**Table 1:** Antiparasitic activity in *L. major-luc* promastigotes, and cytotoxicity to intraperitoneal mouse macrophages (IPΦ) or monkey kidney cells (LLC-MK2) of **5A** and analogs. EC_50_ Median effective concentration. ± values are the estimated EC_50_ interval. CC_50_ Median cytotoxic concentration. ± values are the estimated CC_50_ interval S.I. Selective Index (CC_50_ mammalian cells)/(EC_50_ in *L. major-luc* promastigotes).

Compound	*Leishmania major-luc*	Mammalian Cells	Mammalian Cells
	Promastigotes	IPΦ	LLC-MK2
	EC_50_ (μM)	CC_50_ (μM) [S.I.]	CC_50_ (μM) [S.I.]
**5A**	**~0.3410**	**~ 10.40 [30.50]**	**17.69 ± 1.12 [52.85]**
**5B**	5.98 ± 1.72	N/A	N/A
**5C**	4.73 ± 0.69	N/A	N/A
**5D**	**0.09 ± 0.02**	**27.89 ± 3.19 [310]**	**>80**
**5E**	**0.78 ± 0.11**	**16.59 ± 1.52 [21.27]**	**80 ± 4.45 [102.56]**
**5F**	>12.50	N/A	N/A
**5G**	>12.50	N/A	N/A
**5H**	3.05 ± 0.47	N/A	N/A
**5I**	5.5 ± 1.80	N/A	N/A

**Table 2:** In silico study of **5D** and control Quinacrine Mustard.

Receptor	Ligand	Structure	Glide SP (XP)	IFD XP (IFD Score)
**TryR (2JK6)**	**5D**	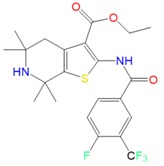	−4.6 (−5.5)	−10.0
	**Quinacrine Mustard (control)**	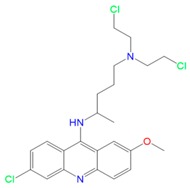	−6.9 (−6.7)	−9.2
